# Can a Shorter Dwell Time Reduce Infective Complications Associated with the Use of Umbilical Catheters?

**DOI:** 10.3390/antibiotics13100988

**Published:** 2024-10-18

**Authors:** Martina Buttera, Lucia Corso, Leonardo Casadei, Cinzia Valenza, Francesca Sforza, Francesco Candia, Francesca Miselli, Cecilia Baraldi, Licia Lugli, Alberto Berardi, Lorenzo Iughetti

**Affiliations:** 1 University of Modena and Reggio Emilia, Via del Pozzo 71, 41224 Modena, Italy; 2 Pediatrics Unit, University Hospital of Modena, 41224 Modena, Italy; 3 Neonatal Intensive Care Unit, University Hospital of Modena, 41224 Modena, Italy

**Keywords:** UVC, CLABSI, central catheters, umbilical catheters, newborn, neonates

## Abstract

Background: Umbilical venous catheters (UVCs) are the standard of care in neonatal intensive care units (NICUs) to administer fluids, parenteral nutrition and medications, although complications may occur, including central line-associated blood stream infections (CLABSIs). However, the dwell time to reduce CLABSI risk remains an open issue. Methods: We performed a single-center retrospective study of newborns hospitalized in the Modena NICU with at least one UVC inserted over a 6-year period (period 1: January 2011–December 2013; period 2: January 2019–December 2021). We selected a non-consecutive 6-year period to emphasize the differences in UVC management practices that have occurred over time in our NICU. The UVC dwell time and catheter-related complications during the first 4 weeks of life were examined. Results: The UVC dwell time was shorter in period 2 (median 4 days vs. 5 days, *p* < 0.00001). Between the two periods, the incidence of CLABSIs remained unchanged (*p =* 0.5425). However, in period 2, there was an increased need for peripherally inserted central catheters (PICCs) after UVC removal, with a rise in PICC infections after UVC removal (*p =* 0.0239). Conclusions: In our NICU, shortening UVC dwell time from 5 to 4 days did not decrease the UVC-related complications. Instead, the earlier removal of UVCs led to a higher number of PICCs inserted, possibly increasing the overall infectious risk.

## 1. Introduction

The catheterization of umbilical vein is an invasive procedure commonly practiced in neonatal intensive care units (NICUs). Umbilical venous catheters (UVCs) are central venous catheters uniquely designed for newborns due to the presence of the umbilical vein, one of three blood vessels located within the umbilical cord [1]. UVCs represent a life-saving medical tool, providing prompt and painless access to the newborn’s venous system to administer fluids, medication and parenteral nutrition and to obtain blood samples [2]. Umbilical vein catheterization is typically performed during the first 48 h postpartum, when the umbilical cord stump is feasible, and the visualization of the umbilical vein is facilitated [3].

However, in the last few years, ultrasonography has emerged as the definitive technique for assessing the precise UVC tip positioning, overcoming the use of thoracoabdominal radiography [4]. Nevertheless, the use of UVCs does not appear to be risk-free, being associated with both mechanical and infectious complications. The first ones include a wide range of adverse events such as malposition (30% of cases), peritoneal extravasation (2.8% of cases), venous thrombosis (1.9% of cases), difficulties in removal (0.4% of cases), symptomatic thrombosis (0.4% of cases), cardiac tamponade (0.1% of cases) and pleural effusion (0.1% of cases) [5].

Among infective complications, central line-associated bloodstream infections (CLABSIs) represent one of the most concerning ones, being potentially lethal, especially for premature infants, but preventable by adopting the correct and most up-to-date bundles of care [6].

In the latest document published in 2024 by the Centers for Disease Control and Prevention (CDC), CLABSIs are defined as a laboratory-confirmed bloodstream infection where an eligible organism is identified and an eligible central line is present. The CDC considers “Eligible Central Line” a line that has been in place for more than two consecutive calendar days; such lines are eligible for CLABSI until the day after removal from the body or patient discharge, whichever comes first [7]. Despite the extensive scientific debate, the maximum UVC dwell time to minimize CLABSI risk remains an open topic. The Infusion Therapy Standards of Practice (INS) guidelines recommend, as an infection prevention strategy, to remove UVCs after 4 days, followed by the insertion of a peripherally inserted central catheter (PICC) if a central line is still needed [8]. Nevertheless, if a PICC is unnecessary, the guidelines suggest limiting the UVC dwell time to 7 to 10 days, to reduce risks of infectious and thrombotic complications [9]. In a document published in 2022, the CDC underscored the limited understanding of the impact of umbilical catheter dwell time on CLABSI, even if several studies suggested that the longer an umbilical arterial or venous catheter remained in place, the higher the infective risk was [10]. However, there are many studies that do not show an association between UVC dwell time and risks of CLABSI, highlighting how this is still a controversial issue [11,12]. Further studies are needed to help clinicians in deciding whether there is a correlation between prolonged UVC dwell time and risks of CLABSI, or to assess how long UVCs may safely remain in place.

We assessed UVC complications in a level 3 NICU where the management of UVCs has changed over time and the focus on early removal has increased. Furthermore, during the same period, the use of umbilical arterial catheters decreased, while the use of empirical antibiotics became increasingly targeted.

We therefore aimed to investigate whether a progressive reduction in the UVC dwell time was associated with less infectious complications. Data were analyzed by comparing two periods (2011–2013 vs. 2019–2021) far apart enough to reveal changes occurring in catheter management practices.

## 2. Results

During the 6-year considered period, 569 newborns of all gestational ages were admitted to our NICU and had at least one UVC inserted (period 1, *n* = 310; period 2, *n* = 259).

### 2.1. Demographic Characteristics of the Cohort

Table 1 shows the demographics of the study population. During period 2, newborns were less likely to be small for gestational age (SGA, i.e., birth weight less than the 10th percentile), while median gestational age at birth increased. Further demographics of the study population are summarized in Table 1.

### 2.2. UVC Dwell Time, Reasons for UVC Removal or Replacement and Simultaneous UAC Placement

Table 2 summarizes the UVC dwell time, the reason for UVC removal (planned or due to complications) and the presence of UACs in the entire cohort in the two study periods. Our data confirm that UVC dwell time significantly decreased from period 1 (median of 5.0 days) to period 2 (median 4.0 days) together with a reduced use of UACs in period 2. Moreover, the number of PICCs inserted after UVC removal had a borderline increase from period 1 to period 2 (*p* = 0.0512). The remaining variables did not change over time.

### 2.3. Analysis of UVC-Related Complications

Table 3 summarizes the UVC-related complications in the two studied periods and in the entire cohort. The incidence of either infectious or mechanical complications remained stable in both periods.

In period 1, 10 cases of sepsis occurred when a UVC was in place. Six of these cases were vertical infections and therefore considered Early-Onset Sepsis (EOS). The remaining four cases were considered, according to the definition, UVC-related: two cases were due to *Staphylococcus epidermidis*, one due to *Staphylococcus aureus* and one due to *Candida albicans*.

By contrast, six cases of sepsis occurred in period 2. One out of six episodes was considered EOS, while five cases of UVC-related infections were documented: two were caused by *Staphylococcus epidermidis*, two by *Staphylococcus aureus* and one by *Enterococcus faecalis*.

Regarding mechanical complications, 19 cases were observed in period 1: 79% (15 out of 19) were due to displacement of the catheter, 5% (1 out of 19) due to extravasation, 5% (1 out of 19) due to thrombosis and 11% (2 out of 19) due to catheter rupture.

The same number of mechanical complications was observed in period 2: 74% (14 out of 19) were due to displacements, 16% (3 out of 19) due to catheter occlusions and 10% (2 out of 19) due to persistent blood leakage.

In addition, 24 and 15 neonates died in period 1 and period 2, respectively; however, none of these deaths were attributed to complications related to UVCs.

### 2.4. PICC-Related Infections

**Figure 1:**
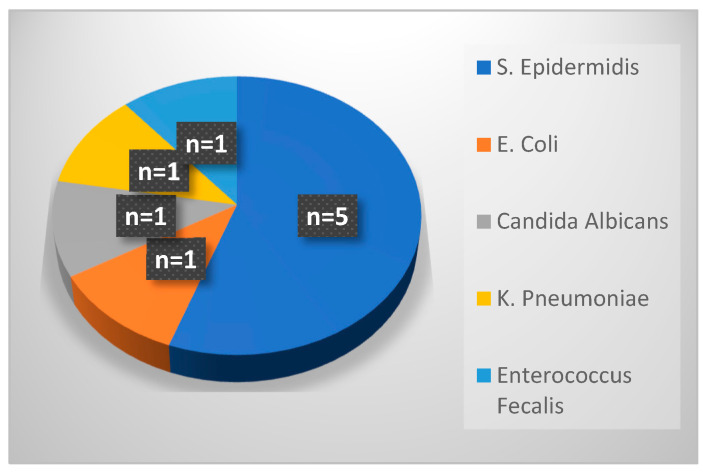
Pathogens responsible for PICC-related infections in period 1.

Figure 1 shows pathogens responsible for PICC-associated infections during period 1. There were nine cases of CLABSIs occurring after UVC replacement with a PICC line: more than half were caused by *Staphylococcus epidermis*, and the remaining by *Escherichia coli*, *Candida albicans, Klebsiella pneumoniae* and *Enterococcus faecalis*. Among nine cases of PIC-related infections, seven occurred in preterm neonates.

**Figure 2:**
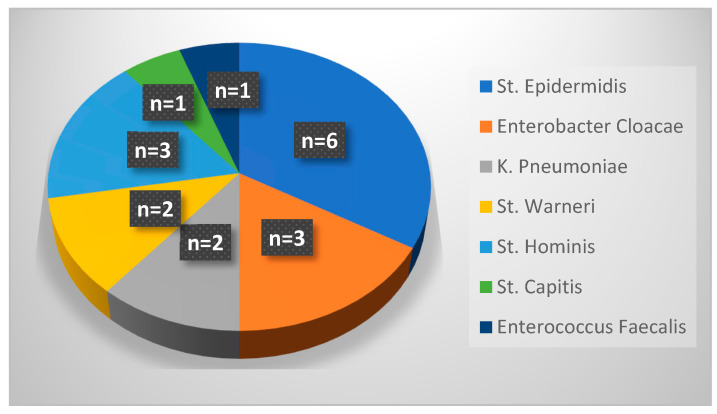
Pathogens responsible for PICC-related infections in period 2.

Figure 2 details the pathogens responsible for PICC-associated CLABSIs during period 2. There were 18 cases of CLABSIs occurring after a UVC was replaced with a PICC: similarly to period 1, the majority of cases were due to infection by *Staphylococcus epidermis*, followed by *Enterobacter cloacae*, *Staphylococcus hominis*, *Staphylococcus warneri*, *Klebsiella pneumoniae*, *Enterococcus faecalis* and *Staphylococcus capitis*. Among 18 cases of PICC-related infections, 17 occurred in preterm neonates.

Table 4 compares the incidence of PICC-related infections in both periods, which increased from period 1 to period 2. Furthermore, the table shows that the total number of central venous catheters (CVCs, which includes UVCs and PICCs) inserted in newborns in the first month of life rises from period 1 (median 1.0) to period 2 (median of 2.0) in the whole population and in premature infants. The comparison of the remaining variables was insignificant.

## 3. Materials and Methods

We conducted a retrospective, single-center study in the NICU of the Policlinic University Hospital of Modena (Modena, Italy). This is a high-volume level 3 facility, with inborn neonates accounting for most admissions. The NICU contains 20 cots and receives approximately 450 admissions per year, and the medical staff consists of 12 physicians. The population of infants on the ward mainly includes premature newborns, with special attention to post-surgery patients, coming from other wards or infants being transferred from other facilities, with surveillance cutaneous and mucosal swabs being taken on admission for inborn and outborn patients alike, and the possibility of cohorting.

Data from newborns admitted to our NICU with at least one UVC inserted during a 6-year period (period 1, from 1 January 2011 to 31 December 2013, and period 2, from 1 January 2019 to 31 December 2021) were retrospectively analyzed. The 2 periods were selected because of changes in the management of UVCs. In period 1, the attention to early UVC removal was low, and some UVCs were left in place for up to 15 days. In contrast, in period 2, clinicians aimed at early UVC removal (usually starting from day 4). Furthermore, umbilical arterial catheters (UACs) were less commonly used (particularly among neonates of higher gestational age), whereas efforts were made to shorten the duration of empirical antibiotic courses and to prevent healthcare-associated infections.

Infants of any gestational age who underwent a UVC placement were included. We included both inborn neonates and newborns referred to our NICU from other centers (outborn) with a UVC in place. For each patient, we examined demographic and maternal risk factors, data regarding UVC removal, infective and mechanical complications and case fatalities.

The NICU computerized medical record (“MetaVision”) was searched to obtain information regarding newborns’ demographics, clinical history, delivery information, reasons for the placement of the central line, characteristics of the removal and the occurrence of any mechanical or infectious complications. In addition, the microbiology database was searched to retrieve any positive isolate that occurred during study periods and pathogens yielded in cultures. The data were anonymously collected by using an Excel file protected by a password.

Analyses were performed using MedCalc version 9.3 (MedCalc Software, Ostend, Belgium). Continuous variables were expressed as medians and ranges. Categorical data were expressed as numbers and percentages. Additionally, the Mann–Whitney rank sum test and χ2 test were used to compare the continuous and categorical variables between groups, respectively. *p* < 0.05 was considered significant.

In this study, we defined CLABSI as a central line-associated bloodstream infection (CLABSI), which is defined as a laboratory-confirmed bloodstream infection, not related to an infection from another site, which develops 48 h after the placement of a central line or within 48 h of its removal [13]. Since the collection of the data began in 2021, the definition of CLABSIs adopted in the study is slightly different from the most recent CDC definition. All infants with CLABSIs included in the study had symptoms that led clinicians to obtain the blood culture. Because CLABSIs are typically considered late infections, infants with early infection (within 48 h of life) were considered to have a vertical transmission of the disease.

## 4. Discussion

We retrospectively studied a large cohort of infants admitted to our NICU during a period of 6 non-consecutive years, comparing two different time frames (2011–2013 and 2019–2021). This broad time range was chosen to better emphasize potential complications following significant changes in UVC management, including variations in dwell time and antibiotic usage.

As mentioned before, the presence of a correlation between UVC dwell time and CLABSI risk is still an open issue in the scientific literature. A recent review has evaluated nine studies assessing the optimal dwell time for UVC replacement in the context of reducing infective risks in newborns [13]. In this review, five studies out of nine analyzed found no evidence of a correlation between catheter-related infectious complications and UVC dwell time [11,14,15,16,17]. Nevertheless, these studies had important limitations, such as a small sample size, retrospective design and lack of randomization. By contrast, four studies found an increased risk of catheter-related infectious complications after a prolonged UVC dwell time [18,19,20,21]. Among these four studies, a large multicenter analysis enrolling 3985 neonates was included, showing that elective early removal of UVC before day 4 and its replacement with a PICC would reduce CLABSI risk [20]. The remaining three studies suggested removing UVCs within 7 days from their placement to reduce complications [18,19,21]. Consistent with these findings, another review conducted by D’Andrea suggested that, when prolonged central access is required, UVCs could be replaced within 4 days [4]. Guidelines from the Infusion Nursing Society also recommend removal within 4 days and its replacement with a PICC, although allowing a maximum UVC dwell time of 7–10 days if no other central access is needed [9]. A recent prospective study including 575 neonates suggests that the UVC should be routinely removed by day 3, although the authors did not consider additional complications of CVCs inserted after UVC removal [22,23].

The current study found that a reduction in UVC dwell time from 5 to 4 days does not decrease the incidence of UVC-associated infective complications, while it could increase the total number of CVCs placed (UVCs or PICCs) within the first month of life and the risk of infectious complications deriving from PICC placement after UVC removal. The reduced dwell time noticed over the years indicates efforts of clinicians in our NICU to reduce both the UVC dwell time and the insertion of UACs, to align with best practices in catheter management. It is not surprising that we did not observe a decreased incidence of infectious complications when reducing UVC dwell time from 5 to 4 days, since the shortening time is minimal. In fact, most studies examining infectious complications related to UVC dwell time typically compare groups of infants with significantly different dwell times, with a study even leaving the UVC in place for up to 28 days [11]. A notable result from our study is that reducing UVC dwell time possibly increased infective complications due to the need for other central access, such as PICCs.

The current study has several limitations. Firstly, due to the retrospective design of the study, some data may have been biased, although the analysis was accurate and information derived from multiple sources. Secondly, the sample size is relatively small, preventing a full reliability of the results, considering that the rate of catheter-related complications is generally low. Moreover, we observed significant changes in the characteristics of the study population from period 1 to period 2, reflecting the different population admitted to the NICU over time. Indeed, the median gestational age increased, while the number of SGA newborns decreased; consequently, the interpretation of our findings should be contextualized within these demographic shifts. Indeed, it is well established from the scientific literature that infants born prematurely and categorized as SGA are inherently more susceptible to a wide spectrum of complications, particularly those associated with infections [24,25,26].

Overall, our reduction in UVC dwell time to 4 days aligns with contemporary best practices and recommendations. Further larger and randomized studies are needed to clarify the optimal UVC dwell time. Moreover, we underscore the need for a comprehensive, patient-centered approach to catheter management.

## 5. Conclusions

Our data do not confirm that shortening dwell time from 5 to 4 days decreases the risk of complications, either infectious or mechanical. However, as evidenced by several studies, the UVC dwell time appears to be a risk factor for CLABSIs, and therefore, the UVC should be promptly removed when no longer needed. Our investigation also reveals an increase in the number of CVC inserted following UVC removal and a possible increase in infectious complications from PICCs. Given that preterm infants are at increased risk of infectious complications, our observation warrants further studies in a larger population.

## Figures and Tables

**Table 1:** Demographics of newborns in period 1 and period 2. Data are presented as median (IQR) or number (%).

	Entire Cohort (*n* = 569)	Period 1 (2011–13) (*n* = 310)	Period 2 (2019–21) (*n* = 259)	Comparison (*p*-Value)
Gestational Age	33 (29–39)	32 (29–39)	35 (29–39)	*p* = 0.04846
Apgar score at 5′	8 (6–9)	8 (6–9)	8 (6–9)	*p* > 0.05
Birth weight	2035 (1158–3180)	1830 (1123.5–3102.5)	2255 (1187.5–3245)	*p* > 0.05
Vaginal delivery	217 (38.1)	108 (34.8)	109 (42)	*p* > 0.05
Term newborn (≥37 weeks)	218 (38.3)	108 (34.8)	110 (42.5)	*p* > 0.05
Late preterm (24–36 weeks)	60 (10.5)	32 (10.3)	28 (10.1)	*p* > 0.05
Very preterm (28–33 weeks)	193 (33.9)	116 (37.4)	77 (29.7)	*p* > 0.05
Extremely preterm (≤28 weeks)	98 (17.2)	54 (17.4)	44 (17)	*p* > 0.05
SGA	103 (18.1)	70 (22.6)	33 (12.8)	*p* = 0.0024
Hypothermia	60 (10.5)	29 (9.3)	31 (12)	*p* > 0.05

SGA = small for gestational age.

**Table 2:** Data regarding umbilical catheters in period 1 and period 2. Data are presented as median (IQR) or number (%).

	Entire Cohort (*n* = 569)	Period 1 (2011–2013) (*n* = 310)	Period 2 (2019–2021) (*n* = 259)	Comparison (*p*-Value)
UVC dwell time (days)	5 (3–6)	5 (4–6)	4 (3–5)	*p* < 0.0001
Any complications (infective/mechanical)	31 (5.4)	14 (4.5)	17 (6.5)	*p* > 0.05
Removal for complications	17 (3)	7 (2.3)	10 (3.9)	*p* > 0.05
UVC replacement with PICC after removal	245 (43)	122 (39.4)	123 (47.5)	*p* > 0.05
Simultaneous UAC	66 (11.6)	47 (15.2)	19 (7.3)	*p* = 0.0037

UAC: umbilical arterial catheter; UVC: umbilical venous catheter; PICC: peripherally inserted central catheter.

**Table 3:** UVC-related complications in period 1 and period 2. Data are presented as a number (%).

	Entire Cohort (*n* = 569)	Period 1 (2011–2013) (*n* = 310)	Period 2 (2019–2021) (*n* = 259)	Comparison (*p*-Value)
All sepsis cases	16 (2.8)	10 (3.2) *	6 (2.3) **	*p* > 0.05
UVC infective complications	9 (1.6)	4 (1.3)	5 (1.9)	*p* > 0.05
UVC mechanical complications	38 (6.7)	19 (6.2)	19 (7.3)	*p* > 0.05
Newborn’s death	39 (6.9)	24 (7.7)	15 (5.8)	*p* > 0.05

UVC: umbilical venous catheter. * Six cases out of ten were vertical infections (five caused by *Escherichia coli*, 1 by *Enterococcus fecalis*); ** one case out of six was a vertical infection caused by *Staphylococcus epidermidis.*

**Table 4:** Variables regarding umbilical venous and peripherally inserted central catheters in period 1 and period 2. Data are presented as median (IQR) or number (%).

	Entire Cohort (*n* = 569)	Period 1 (2011–2013) (*n* = 310)	Period 2 (2019–2021) (*n* = 259)	Comparison (*p*-Value)
Median number of CVCs during 1st month of life in the whole population	1 (1–2)	1 (1–2)	2 (1–2)	*p* = 0.00336
Median number of CVCs in preterm neonates during 1st month of life	2 (1–2)	2 (1–2)	2 (1–3)	*p* = 0.00042
Median number of CVCs in full-term neonates during 1st month of life	1 (1–1)	1 (1–1)	1 (1–2)	*p* > 0.05
PICC infections after UVC removal	27 (4.7)	9 (2.9)	18 (6.9)	*p* > 0.05

UVC: umbilical venous catheter; CVC: central venous catheter.

## Data Availability

The raw data supporting the conclusion of this article will be made available by the authors on request.
